# Analysis and exploration of infertility policies in Iran: a study protocol

**DOI:** 10.1186/s12961-019-0505-3

**Published:** 2020-01-15

**Authors:** Bahar Morshed-Behbahani, Minoor Lamyian, Hassan Joulaei, Ali Montazeri

**Affiliations:** 1 Department of Reproductive Health and Midwifery, Faculty of Medical Sciences, Tarbiat Modares University, Tehran, Iran; 2 Health Policy Research Center, Institute of Health, Shiraz University of Medical Sciences, Shiraz, Iran; 3 Population Health Research Group, Health Metrics Research Center, Institute for Health Sciences Research, ACECR, Tehran, Iran; 4 Faculty of Humanity Sciences, University of Sciences & Culture, ACECR, Tehran, Iran

**Keywords:** Infertility, policy analysis, Iran

## Abstract

**Background:**

Infertility is a complex and neglected reproductive health issue of global proportions, with varying effects on couples and their relationships. Therefore, international organisations and several countries have been compelled to consider infertility policies. In recent years, a shift in population policy trends toward increasing birth rates in Iran have set infertility policies in the health sector’s agenda. Since infertility and its associated problems are multifactorial, all health systems, including that of Iran, need to have a comprehensive policy package that covers all of its dimensions. Policy analysis is necessary to formulate such policies. This project will therefore analyse the infertility policies in Iran’s health sector and clarify the multilateral effects of their different components.

**Methods:**

This multidisciplinary study outlines the conceptual framework of infertility policies and consists of three stages. Stage I will involve the review of infertility policies in selected countries and Iran for the operational model of infertility programmes, rules and policies. Stage II will consist of a documentary infertility policy analysis of Iran. At this stage, Iran’s infertility policies will be analysed using the Walt and Gilson framework in four areas, namely content, context, process and actors. Stage III will involve the analysis of infertility policies in Iran. At this stage, a qualitative study will be conducted to understand and provide in-depth explanations of the existing policies. Finally, the concepts and outcomes obtained from the first stage will be combined with the content of the qualitative analysis of the second and third stages for exploration of Iran’s infertility policies, and a package including a framework for infertility policies will be proposed.

**Discussion:**

The findings of this study can be used by the Ministry of Health and public health policy-makers to determine which policies, in view of socio-cultural and economic contexts and actors’ roles in each country, can be used to reach the goals defined by international organisations, on the prevention of infertility and reproductive health.

## Background

Infertility is a complex and neglected reproductive health issue worldwide [1]. Globally, more than 186 million people suffer from infertility [2], with various effects on couples and their relationships [3]. It evidently makes couples feel desperate, worried, indignant, inadequate, guilty and socially isolated. It is believed that the negative consequences of social exclusion due to infertility could lead to divorce, violence against women, shame and social suffering [4, 5]. In addition, in many countries, infertility treatment poses an extra burden to couples and families [6, 7]. As such, WHO recommended to its country members the establishment of policies for infertility treatment [8] and, in a report, asked that health systems gradually shift from therapeutic management to prevention as needed [9]. Indeed, the Human Reproduction Programme developed new guidelines for infertility care to help developing countries integrate policies into existing services, systems and reproductive health programmes [10].

An assessment of existing policies for infertility in different countries highlights that the oldest guidelines belong to the United Kingdom and the United States of the America. In the United Kingdom, the related services, costs and insurance have been codified in the National Health Services. The protocols, rules and guidelines are evaluated and reviewed annually by the National Institute for Health and Care Excellence [11–13]. In the United States, the American Society for Reproductive Medicine, the United States Department of Health and Human Services, and the Center for Disease Control and Prevention also formulated the required policies and guidelines and defined infertility services at three levels — early assessment, prevention, and early and specialised treatment [14–17].

The prevalence of infertility in Iran ranges from 10.3% to 24.9% [18, 19]. Similar to any other country, complications of infertility for Iranian couples include psychological distress [20] and social suffering [3, 21]. Unfortunately, financial problems, such as expensive treatments and lack of insurance coverage, are the major worries of infertile couples [3] and thus treatment drop-out in Iran is as high as 28.3% [22].

The abovementioned reasons, on the one hand, and shifting in population policy in order to increase birth rate in Iran [23], on the other, have been sufficient to set infertility policies within the agenda in recent years in the health system of Iran.

To the best of our knowledge, current policies for infertility in Iran are incoherent, exclusively therapeutic [7] and distributive [24]. These policies have led to the establishment of public and private healthcare centres with inappropriate distribution [1]. Other infertility policies were developed with the goal of encouraging and increasing fertility replacement levels. However, these caused financial difficulties in the provision of drugs and infertility treatment for all infertile couples, regardless of their financial condition [7]. These policies did not address issues such as prevention, early diagnostic and referral processes, primary and supportive treatments, and accessibility of infertility services. Even in the latest healthcare reform plan infertility services have not been included.

Since infertility and its associated problems are multifactorial in nature, so all health systems, including that of Iran, need to have a comprehensive policy package that covers all of its dimensions. To formulate such policies, a through policy analysis is necessary. Indeed, policy analysis will help to examine the role of four elements — actors, process, context and content. Such analysis might specify and predict the causes of the success or challenge, barriers or facilitators of policies, and required planning to improve them [25]. In this regard, it is important to know how these four factors affect the infertility policies and how they have been successfully implemented.

Therefore, this study is designed to analyse the infertility policies in Iran’s health sector and clarify the multilateral effects of different components of these policies, including content, context, process and actors on running these policies. Since infertility policies are either new or an ongoing process in most countries, this study might contribute to existing knowledge on the topic and help policy-makers in order to formulate a plan in health systems.

## Objectives

The main goal of this study, which is planned to last 2 years, is to analyse and explore infertility policies in Iran’s health system. Analysis will cover prevention, diagnosis, timely treatment and supportive policies, while exploration will focus on proposing the required policies. Thus, the objectives are indicated as follows:

1. To review and compare infertility programmes in Iran and other countries.
2. To analyse existing policies for infertility in Iran.
3. To propose a holistic package of prevention, therapeutic and supportive infertility policies for Iran.

## Methods/Design

This multidisciplinary study outlines the conceptual framework of infertility policies and consists of the following three stages.

### Stage I: Review of infertility services policy in selected countries and Iran

In this stage, a systematic literature review on infertility policies guided by the Preferred Reporting Items for Systematic Reviews and Meta-analyses (PRISMA) will be performed [26].

#### Definition

The policy documents will be defined as all formal records and reports that were written by national governments, national scientific communities and academic societies, national authorities and international organisations’ decisions, reports, plans and actions from WHO or World Bank, world health statistics, world development indicators, and demographic and health surveys. The type of evidence will include provincial annual reports, core public health function/standards documents, health human resources, human resource planning annual reports, business plans, commissioning policy documents, clinical guidelines, health profession legislation, and other public health reports such as competency development and leadership frameworks.

#### Search engines and time period

Documents will be selected from 1994 (since the Cairo Conference) until the end of 2018. The Cairo Conference recommended countries to plan and implement action to prevent and treat infertility [27]. The search engines will include PubMed, ISI, Google Scholar, all public websites, websites of health ministries, and websites of infertility clinics searching for review articles, grey papers, government records and guidelines, protocols, and clinical guidelines.

#### Search strategy

The search strategy will include a combination of the keywords ‘infertility’, ‘policy-making’, ‘affordability’, ‘availability’, ‘acceptability’, ‘awareness’, ‘responsibilities’, ‘insurance’, ‘health policy’, ‘prevention’, ‘financial management’, ‘childlessness’, ‘equity’, ‘utilization’ and ‘cost’.

#### Inclusion and exclusion criteria

Documents in English language that address the operational model of infertility programmes, rules and policies will be included. The main focus at this stage will be on infertility policies, executive processes and prevention or implementation of policies in providing fair services at three levels (prevention, early treatment and supportive care). Irrelevant documents will be excluded. For instance, discussion papers, advertisements, video clips, newspapers, online advertising sites, movie content, and marketing channels will not be included.

#### Selection of countries

The selection of countries will be based on the availability of appropriate documents and existence of comprehensive infertility policy from all five continents and from all income groups (high, middle and low) with the help of the expert panel and the research team.

#### Data extraction and synthesis

First, a datasheet for each country will be prepared including the following information: name and aim of the policy, author(s) or organisation name and actors involved; this information will be tabulated and made ready for further analysis (Table 1). We will then identify three components for each policy document as defined by universal health coverage, as follows: (1) financial protection, (2) population coverage, and (3) service package, including services for prevention, treatment and supportive care [28]. The indicators and practical definition of policy components were provided and were finalised in two sessions by an expert panel consisting of two specialists in health policy, two public health scientists, a gynaecologist and a reproductive health specialist. These explanatory variables and indicators are described in Table 2. The findings will be then be scored; for each component, if the policy satisfies the condition, a score of 3 will be assigned, otherwise, a score of 2 (intermediate) or 1 (low) will be considered (Table 3). Finally, a scoring sheet containing scores for all countries, including Iran, will be provided and compared. A hypothetical scoring sheet is provided in Table 4.

**Table 1:** A schematic view of summary of each country’s profile

Country	Title	Year	Name of the policy	Aim(s) of the policy	Author(s) or organisation name	Actors involved
.

**Table 2:** Definitions of dependent and explanatory variables

Variable	Measure
Financial protection	
Insurance coverage	The amount of risk or liability that is covered for an individual or entity by way of insurance infertility services
Government funding	Money provided by the government to pay for infertility services
Supply of voluntary and charitable donations	Financial support from voluntary associations or non-governmental organs
Population coverage	
Gender	Gender sensitivity in the service delivery^*^
Age	Availability of services for all age
Urban/rural coverage	Availability of infertility services in urban and rural areas
Service package	
Acceptability	Cultural, social and religious acceptance of infertility services
Accessibility/availability	Considering the number of service centres in relation to the population and their distribution
Awareness/registry	Having proper registry system
Preventive	Existence of a preventive service, which includes check-ups, patient counselling and screenings to prevent infertility
Diagnostic and curative	Providing all infertility diagnostic and curative services
Rehabilitation, and supportive care	Existing rehabilitative infertility care includes empowerment of the couples to manage their conditions with proper counselling and enabling them to enjoy life by appropriate rules for adoption

* 1. Availability of infertility services to single women or men ,widows, and homosexual. 2. Equal treatment (e.g.,waiting time,courtesy, privacy, information given) for male and female clients

**Table 3:** Indicators that will be used for scoring

Score	1 (Low)	2 (Intermediate)	3 (Good)
Financial protection			
Insurance coverage/government funding/supply of voluntary and charitable donations	One of the items	Two of the items	All three items
Population coverage			
Gender	Only for female or male	Married female and male	All
Age	Limited	Reproductive age period	Not limited
Urban/rural coverage	Urban only without rural access	Urban with difficult rural access	Urban with good rural access
Service package			
Acceptability	Low	Moderate	High
Accessibility/availability	Low	Moderate	High
Registry	Low	Moderate	High
Preventive services	Lack of policy	Poor policies	Efficient policies
Diagnostic and curative services	Lack of policy	Poor policies	Efficient policies
Rehabilitation and supportive care services	Lack of policy	Poor policies	Efficient policies

**Table 4:** Scoring sheet for infertility policy based on universal health coverage framework

Economic situation	Low income		Middle income		High income	
Country	a	b	c	d	e	f
Financial protection						
Insurance coverage/Government funding/supply of voluntary and charitable donations						
Population coverage						
Gender						
Age						
Urban/rural coverage						
Service package						
Acceptability						
Accessibility/availability						
Registry						
Preventive services						
Diagnostic and Curative services						
Rehabilitation and supportive care services						
Total score*						

* Total score can range from 10 (least) to 30 (highest)

### Stage II: Documentary analysis of Iranian infertility policies

During this stage, a review of the actual published policies and guideline documents in Iran will be carried out [29, 30]. As such, we will use the content analysis. It is suitable for exploring rationales, strengths and weaknesses of the infertility policies [31, 32]. This will help us to analyse content, context, process, and actors that are prominent or have a role in these policies. We will code all the data that extracted at stage I, and the rest of the documents related to the infertility care policies in Persian language. Thematic analysis will then be conducted and categorised based on the Walt and Gilson framework [33].

### Stage III: Infertility policy analysis of Iran

At this stage, Iran’s infertility policies will be analysed using the Walt and Gilson framework in four areas, namely content, context, process and actors. Infertility policies can include different rules, framework actions to achieve specific goals, action plans, tasks, or an unwritten ethical or cultural code that guides behaviour. Each culture and social context will have different and proprietary health policies. The policies are developed in a variety of ways and have different executive formation processes influenced by different people with different actions [34]. Therefore, all of these will be considered in the policy analysis and exploration of the new infertility policies. In this way, we will achieve the second goal of the study. Thus, a qualitative study will be conducted to understand and provide in-depth explanations of the existing policies.

#### Data sources and sampling

Participants in this section include experienced/most informed experts, including policy-makers and decision-makers involved in fertility and infertility health programmes at the Ministry of Health, and stakeholders including heads of hospitals, insurance agencies, infertility specialists and infertile couples. To select participants, the purposive sampling method and a maximum diversity approach will be used. Inclusion criteria are the job position, the knowledge and awareness, and the sufficient motivation to collaborate in the research. Individual semi-structured interviews will be used to produce data. The interviews will be based on the study objectives. Sampling will continue until data saturation.

#### Rigor

To ensure validity and reliability, the research team will compare and review the initial codes frequently with the initial summaries of the data. The codes will then be provided to the participants in order to be validated. To ensure the validity and reliability of the data, the four criteria of Lincoln and Guba will be used [35].

#### Analysis

Following interviews, coding and analysis will be carried out manually with the framework analysis. This is a qualitative method that is aptly suited for applied social policy research and is currently used for health policy studies. It is adapted to research that has specific questions, a pre-designed sample and a priori issues [36].

### Exploration

In this stage, the policies resulting from the first stage will be integrated with the findings from qualitative content analysis in the second and third stages to synthesise a comprehensive proposed model for infertility prevention and care in Iran by the expert panel. The probable integration model of stages and hypothetical conditions are shown in Table 5 [37].

**Table 5:** Hypothetical conditions

	Finding from stage 1	Finding from stages 2 and 3	Expected results
Condition 1	Policy achievements	The same achievements	Confirmation
Condition 2	Policy achievements	No achievement	Need for development and customisation of policies
Condition 3	No achievement	Achieved policies	Improvement and standardisation of policies

## Discussion

Findings from this policy analysis will underline the complex nature of the policy-making process and the multiple influences and actors over this process. This analysis will be organised around a description of gaps or confirmation of desirable infertility policies in selected countries and national policies in Iran. Findings from this study will contribute to an understanding of the complexities in the roles of actors on the process of policy-making and implementation of policy with considerable social, cultural and health system challenges. This study will improve decision-makers’ insight into the importance of the underlying factors in the formulation of policies, the stakeholders’ contribution to creating a policy prioritisation process, and the state of evidence and documentation in decision-making and policy innovation. The results of this study will also clarify how and why some evidence affects the flow of decision-making regarding fertility programmes and why some problems persist [38]. Increasing understanding of the role of facilitators and barriers to implementing infertility policies for decision-makers, in turn, can lead to the development of processes for accelerating and maximising expected results [39]. Using this method and developing the concepts of the theoretical part based on qualitative procedures, this study will provide new knowledge about the type and quality of evidence, thus providing the necessary evidence to change the perspective of different decision-makers. This change of perspective includes groups or contexts that may normally be less variable, such as medical professionals [40], or may be appropriate where there is a need to make major changes to multiple systems involving multiple organisations and/or different stakeholders.

The findings of this study can be used by national health policy-makers to determine which policies, in view of political, socio-cultural and economic contexts, can be applied to reach the goals defined by international organisations to improve the prevention and care of infertility programmes in Iran. Additionally, exploration of prevention policies and determining the pathway for the referral and treatment of infertile couples can improve reproductive health in at-risk individuals and promote their mental health, social and family status, as well as reducing the catastrophic costs due to infertility care.

## Data Availability

The data will be available on reasonable request from the corresponding author in due course.
